# An eight-year old girl with fever and rash: What is the possible diagnosis?

**DOI:** 10.51866/tyk.298

**Published:** 2023-10-05

**Authors:** Samat Farhani, Miptah Hayaatul Najaa, Su Eng Hu

**Affiliations:** 1 MD, MMed, Tanjung Karang Health Clinic, Kuala Selangor, Selangor, Malaysia. Email: hani5204@gmail.com; 2 MBBCh BAO, MMed, Department of Primary Care Medicine, Faculty of Medicine, Universiti Teknologi MARA, Selayang Campus, Jalan Prima Selayang 7, Batu Caves, Selangor, Malaysia.; 3 MBBS, Lanang Health Clinic, Lor Lanang Barat 7, Pekan Sibu, Sibu, Sarawak, Malaysia.

**Keywords:** Cutaneous lupus erythematosus, Skin rash, Connective tissue diseases

## Abstract

In this clinical challenge, we describe the case of a previously healthy 8-year-old girl who presented to a primary care clinic with fever, reduced oral intake and malaise on day 3 of her illness. Clinical examination revealed that she was tachypnoeic and tachycardic. An erythematous rash was found across the bridge of her nose and cheeks, and several painless ulcers were noted in the oral cavity. Blood investigation showed thrombocytopenia, while urinalysis revealed microscopic haematuria and proteinuria. Useful initial diagnostic imaging studies were discussed, including bedside ultrasound in the ambulatory care setting. It is imperative that primary care providers be vigilant when encountering cases like this.

## Case summary

**Figure 1:**
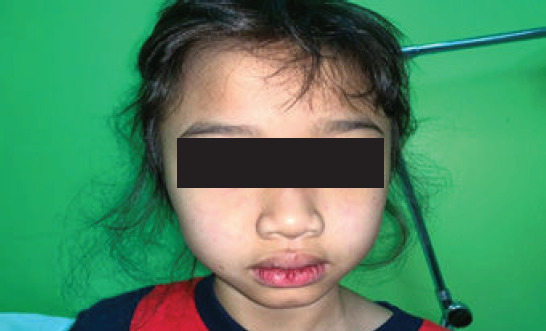
Erythematous rash on the face with a butterfly-like distribution (malar rash).

**Figure 2:**
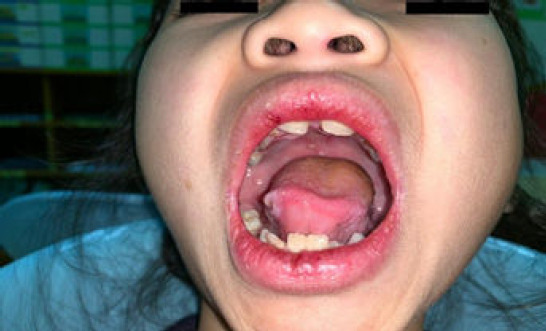
Several ulcers in the oral cavity.

An 8-year-old Malay girl with no known medical illness presented to a primary care clinic with fever (37.9°C), chesty cough, reduced oral intake and sluggishness for 3 days. She had neither headache nor joint pain. Further, she was tachypnoeic (respiratory rate of 35 breaths per minute) and tachycardic (heart rate of 130 beats per minute), with a saturation level of 95% under room air. Physical examination revealed an erythematous flat rash across the bridge of the nose and cheeks, which spared the nasolabial folds (Figure 1), and several painless ulcers on the tongue and palate (Figure 2). Respiratory examination showed dullness on percussion at both lung bases, with reduced air entry bilaterally. No cervical lymphadenopathy was noted. Abdominal examination demonstrated mild generalised tenderness, with no hepatosplenomegaly. A full blood count was conducted, which revealed a platelet count of 121,000/L, indicating thrombocytopenia, and normal haemoglobin level (12.3 g/dL) and leucocyte count (7200/L). Dengue serology and NS1 tests as well as examination of blood films for malaria parasites yielded negative findings. Urinalysis showed the presence of erythrocytes (4+) and protein (4+) in the urine.

**Questions:**

1. What is the most likely diagnosis?
2. What other initial radiological investigations can be performed?
3. What is the management plan?
4. What are the complications that may arise from this condition?

## Answers

1. The most likely diagnosis is juvenile systemic lupus erythematosus (SLE). This diagnosis is supported by the patients sex, as juvenile SLE is mostly predominant in female patients, as well as by the presence of fever with malar rash, oral ulcers with thrombocytopenia and unexplained haematuria and proteinuria. The differential diagnosis includes infectious disease (e.g. dengue, malaria or leptospirosis), malignancy (e.g. leukaemia or lymphoma) or autoimmune disease (e.g. antiphospholipid syndrome).
2. The initial radiological investigation that can be performed to detect pneumonia, pleural effusion or globular heart disease is chest radiography. Chest radiography can be employed to assess pneumonia characteristics, including hilar haziness, prominent vascular marking and areas of consolidation. Additionally, pleural effusion with blunting of the costophrenic angle and ascites can be evaluated. Bedside ultrasound can also be performed for identifying serositis in cases of small effusion. Pericardial effusion is commonly observed as a hypoechoic area surrounding the heart during ultrasound imaging. In cases wherein effusion exceeds 200 mm, it becomes detectable on a plain radiograph.
3. The child should be managed by a multidisciplinary team, ideally consisting of a primary care physician, paediatrician, intensivist and rheumatologist. Once the diagnosis is confirmed, the child should be managed with intravenous steroids for rapid disease control and immunosuppressive drugs for glomerulonephritis manifestation. Concomitant control of peripheral oedema and proteinuria, along with nutritional support, is vital. Cyclophosphamide, an alkylating agent, is reserved for the most severe and life-threatening symptoms.
4. The potential complications include infection and macrophage activation syndrome (MAS). Secondary infections are frequently associated with disease flares in patients in an immunocompromised state. Bacterial infections are the most common types, and intravenous antibiotics should be administered. MAS reflects an uncontrolled immune activation that infiltrates multiple organs such as the spleen, liver and brain, and the symptoms mimic disease flares.

Intravenous immunoglobulin is important during the initial presentation of MAS.

## Case progress

The patient was referred immediately to a paediatrician in the hospital. Chest radiography showed a blunted costophrenic angle at the right lung, with increased vascular marking; no cardiomegaly was noted. Bedside ultrasound revealed mild pericardial effusion associated with bilateral mild pleural effusion and ascites. Further tests revealed negative Coombs test results, low complementary factor (C3 and C4) levels, positive anti-nucleic antibody results (titre=1280, homogeneous) and positive doublestranded DNA antibody results (titre>1000 IU/mL). These findings yielded a diagnosis of juvenile SLE with lupus nephritis, hypertension and severe pneumonia. The patient was started on oral hydroxychloroquine 200 mg once daily, intravenous albumin 20% and intravenous antibiotics for severe pneumonia upon admission. During hospitalisation, a pulse of intravenous methylprednisolone was given for 3 days, followed by a cycle of intravenous cyclophosphamide. In the ward, she also developed hypertension, with blood pressure readings ranging from 115 to 148 mmHg systolic and 87 to 113 mmHg diastolic. She was admitted for a total of 23 days and discharged well with oral prednisolone, hydroxychloroquine and antihypertensive agents.

## Discussion

Juvenile SLE is a rare disease that is more common in Southeast Asian countries than in Western countries.^1^ In Malaysia, the prevalence of SLE has been reported to be 43 per 100,000 people, with children and adolescents comprising 15–20% of this affected population.^1,2^ In general, SLE rarely occurs before the age of 10 years and predominantly affects female patients. In Malaysia, the median age of onset is 10.8 years.^1,3^ Nevertheless, the diagnosis of juvenile SLE is often delayed because it presents with a variety of non-specific symptoms and signs. The present case study provides an example of how a greater sense of awareness and clinical skills are needed by primary care doctors when dealing with children who present with undifferentiated fever and significant clinical findings of SLE.

The literature shows that most patients diagnosed with juvenile SLE fulfil four or more of the American College of Rheumatology classification criteria for SLE.^4^ These criteria can be used as guidance in clinical practice.

The present patient fulfils five of the criteria, including malar rash, oral ulcers, haematological manifestation (thrombocytopenia), renal disorder (marked proteinuria) and serositis (based on ultrasound findings). These findings indicate that primary care doctors should be more vigilant, as substantial delay in the diagnosis of juvenile SLE can lead to serious morbidity and mortality with respect to secondary infection or MAS.^4^
